# Not Only Glycaemic But Also Other Metabolic Factors Affect T Regulatory Cell Counts and Proinflammatory Cytokine Levels in Women with Type 1 Diabetes

**DOI:** 10.1155/2017/5463273

**Published:** 2017-05-03

**Authors:** Katerina Stechova, Jana Sklenarova-Labikova, Tereza Kratzerova, Pavlina Pithova, Dominik Filipp

**Affiliations:** ^1^Department of Internal Medicine, 2nd Medical Faculty, Charles University and University Hospital Motol, Prague, Czech Republic; ^2^Department of Paediatrics, 2nd Medical Faculty, Charles University and University Hospital Motol, Prague, Czech Republic; ^3^Department of Physiology, 2nd Medical Faculty, Charles University, Prague, Czech Republic; ^4^Laboratory of Immunobiology, Institute of Molecular Genetics of the ASCR, Prague, Czech Republic

## Abstract

Type 1 diabetic (T1D) patients suffer from insulinopenia and hyperglycaemia. Studies have shown that if a patient's hyperglycaemic environment is not compensated, it leads to complex immune dysfunctions. Similarly, T1D mothers with poor glycaemic control exert a negative impact on the immune responses of their newborns. However, questions concerning the impact of other metabolic disturbances on the immune system of T1D mothers (and their newborns) have been raised. To address these questions, we examined 28 T1D women in reproductive age for the relationship between various metabolic, clinical, and immune parameters. Our study revealed several unexpected correlations which are indicative of a much more complex relationship between glucose and lipid factors (namely, glycosylated haemoglobin Hb1Ac, the presence of one but not multiple chronic diabetic complications, and atherogenic indexes) and proinflammatory cytokines (IL-1alpha and TNF-alpha). Regulatory T cell counts correlated with HbA1c, diabetic neuropathy, lipid spectra parameters, and IL-6 levels. Total T-helper cell count was interconnected with BMI and glycaemia variability correlated with lipid spectra parameters, insulin dose, and vitamin D levels. These and other correlations revealed in this study provide broader insight into the association of various metabolic abnormalities with immune parameters that may impact T1D mothers or their developing child.

## 1. Introduction

Type 1 diabetes (T1D) is a T-helper 1- (Th1-) mediated autoimmune disease that is characterized by a lack of insulin due to autoimmune destruction of pancreatic beta cells [1]. T1D develops more often in young individuals and, at the time of diagnosis, is typically associated with an acute complex metabolic disturbance in the absence of microvascular or macrovascular chronic diabetic complications. In this sense, if diabetes compensation gauged through the levels of glycosylated haemoglobin (HbA1c) is appropriate, T1D patients are relatively healthy people with normal body weight. However, after several years of disease progression, their health gradually worsens and chronic diabetic complications may occur. In fact, they gradually become biologically older, with more apparent health impairment than what is typical for their age-related peers [2]. Overweight and obesity also continue to be more prevalent among individuals with T1D. These conditions are related to worsened insulin sensitivity which further increases the risks of comorbidities, such as metabolic syndrome, and leads to further development and deterioration of T1D-associated microvascular and macrovascular diseases [3]. T1D patients may also suffer from additional autoimmune diseases (the most common is thyroid gland autoimmunity) due to the inclination of their immune system to autoreactivity [4]. Taken together, T1D in a long term represents a multifactorial pathogenic continuum rather than a single-disease entity of autoimmune origin.

In our previous study, we analyzed the impact of maternal diabetes on the immune responses of newborns. Notably, we evaluated the effect of maternal glycaemic control on the cytokine profile of newborn cord blood mononuclear cells (CBMCs). The result of this study demonstrated that nonstimulated or diabetogenic autoantigen-stimulated newborn CBMCs of T1D mothers with poor glycaemic control exhibited lower cytokine and chemokine production compared to those of mothers with well-stabilized glycaemic control [5]. Consistent with this conclusion was the demonstration that high glucose concentration in culture media that mimicked “hyperglycaemia” also dampened the production of cytokines and chemokines [5]. This study not only contributed to our increasing knowledge on foetal cell reprogramming and the importance of an intrauterine environment for the future health status of the child [6], but it also raised important questions concerning the impact of other metabolic disturbances on the immune system. Specifically, we asked whether, on top of glucose metabolites, other metabolic and clinical parameters, such as lipid spectra, atherogenic indexes, vitamin D levels, and hypertension, can exert their impact on critical humoral and cellular components of the immune systems, such as proinflammatory cytokines and Treg counts, respectively. This impact could also potentially involve the interplay between the production of advanced glycation end products (AGE) and the development of chronic diabetic complications.

To provide an initial insight into these inquiries, we selected a group of T1D women in reproductive age who eventually planned to become pregnant in the future. Our study revealed several unexpected correlations which are indicative of a much more complex relationship between glucose and lipid metabolites with elements of the immune system. These results thus provide a more complex view on the interplay between the metabolic state of T1D female patients and the immune parameters that may affect a developing child.

## 2. Material and Methods

### 2.1. Study Subjects and Ethics

We examined 28 nonpregnant T1D women. Their characteristics are summarized in Table 1. Metabolic characteristics of the group studied are described in Supplementary Table 1 available online at https://doi.org/10.1155/2017/5463273. Ethical approval for this study was obtained from the local ethics committee and written informed consent was obtained from all study participants.

### 2.2. Cell Isolation and Flow Cytometry

Peripheral blood mononuclear cells (PBMCs) were isolated from 20 to 25 ml of peripheral blood samples via Ficoll density gradient centrifugation (Amersham Biosciences, Uppsala, Sweden). According to the recommendation of the international working group of the experts in flow cytometry [7], CD3, CD4, CD25, CD127, and FoxP3 markers were used to analyze human Treg cells using a gating strategy illustrated in Supplementary Figure 1. Specifically, CD3-FITC, CD45-PerCP, CD127-APC (Miltenyi Biotec, Bergisch Gladbach, Germany), CD4-Alexa Fluor® 700, (EXBIO a.s., Prague, Czech Republic), Foxp3-PE, and CD25-Brilliant Violet (BioLegend, San Diego, USA) antibodies (Abs) were used. For intracellular staining, we used Foxp3-Fix/Perm and Foxp3-Perm Buffers (BioLegend, San Diego, USA). The flow cytometric data were collected on a LSR II flow cytometer (BD Biosciences, Franklin Lakes, NJ, USA) and analyzed using Summit 4.3 software (Dako, Glostrup, Denmark).

### 2.3. Measurements of Cytokine, AGE, and Protein Levels

Serum levels of cytokines and advanced glycation end products (AGE) were measured using the Instant ELISA kit for IL-1alpha, TNF-alpha, and IL-6 (eBioscience, San Diego, USA) and OxiSelect AGE ELISA kit (Cell Biolabs, San Diego, USA). Protein concentrations were determined with a Pierce™ BCA Protein Assay Kit (Bio-Rad, California, USA).

### 2.4. Routine Biochemical Laboratory and Calculated Metabolic Parameters

To express insulin resistance, the whole-body estimated glucose disposal rate (eGDR) was calculated using the formula

(1) $$\begin{array}{c} eGDR\;\left(mg{\cdot kg}^{-1}{\cdot \min}^{-1}\right)=24.31-\left(12.22\times WHR\right)-\left(3.29\times HTN\right)-\left(0.57\times HbA1c\right), \end{array}$$

where WHR indicates the waist-hip ratio and HTN is the presence or absence of hypertension (expressed as 1/0 in the calculation) [8]. The glycosylated haemoglobin HbA1c, native lipid laboratory parameters, and vitamin D levels were determined using routine methods of the hospital biochemical laboratory. We calculated the risk indexes, that is, the plasma atherogenic index (PAI) as log(triglycerides/HDL cholesterol) which is recommended to be below 0.1 in women, the atherogenic index (AI) as the total cholesterol/HDL cholesterol where AI < 0.1 means the lowest cardiovascular risk, AI ≥ 0.1 ≤ 0.21 as the intermediate risk, and AI > 0.21 as the highest cardiovascular risk for the patients. Remnant cholesterol was calculated as the total cholesterol minus HDL cholesterol minus LDL cholesterol [9]. Glycaemic variability was calculated from patient glycaemia which was measured by the patients glucometer or by CGM (continuous glucose monitoring) during one month before blood sampling.

### 2.5. Statistics

The statistical analyses were performed using SPSS 22.0 for Windows (SPSS Inc., Chicago, IL, USA). The data was examined for normality and nonparametric tests, including nonparametric correlations (Spearmann's coefficient *R* was calculated) that were applied. We used several grouping variables (Table 2) to test the importance of different metabolic factors. For comparisons across groups, we used the Mann-Whitney *U* test for 2 samples and the Kruskal-Wallis 1-way ANOVA for *k* samples. Significance was defined at *p* < 0.05.

## 3. Results

### 3.1. Inflammatory Cytokines

Evaluating relationships between various clinical and biochemical parameters, we found significant correlations between the production of inflammatory cytokines and the level of HbA1c, presence of chronic diabetic complications, and calculated lipid risk indexes.

**Figure 1:**
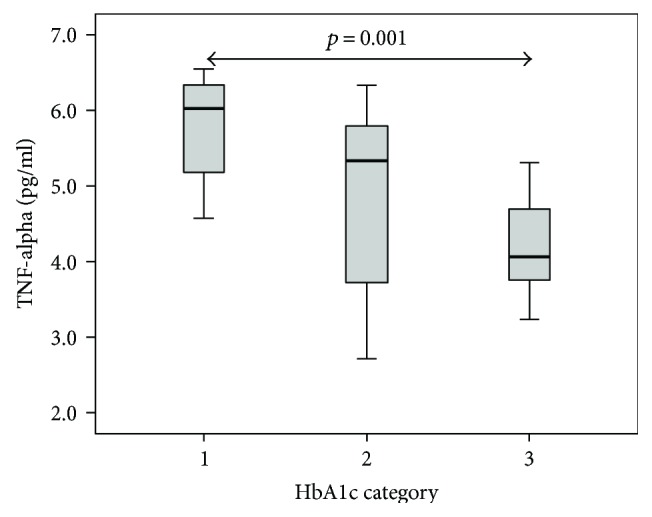
Inflammatory cytokines and HbA1c category. Patients with the worst diabetes stabilization (evaluated according to glycosylated haemoglobin HbA1c) had surprisingly the lowest level of proinflammatory cytokine TNF-alpha. There was a significant difference between this subgroup (marked as category 3) and the subgroup of patients with the best HbA1c (marked as category 1).

Towards this end, T1D patients were split into three groups according to HbA1c values. Group 1 consisted of eight patients who exhibited perfect or acceptable T1D stabilization with HbA1c below 7.5% DCCT. Twelve patients who were classified as not well stabilized represented group 2 with HbA1c in the range of 7.5 to 9% DCCT. Finally, eight patients with HbA1c values over 9% DCCT, indicating a very bad compensation, represented group 3. When we correlated cytokine levels within these three groups (Figure 1), unexpectedly, the lowest TNF-alpha production was found in group 3 which significantly differed from that in group 1 (*p* = 0.001) and even showed tangible but not significant difference with that in group 2. No correlation between HbA1c and IL-1alpha nor with IL-6 was found.

**Figure 2:**
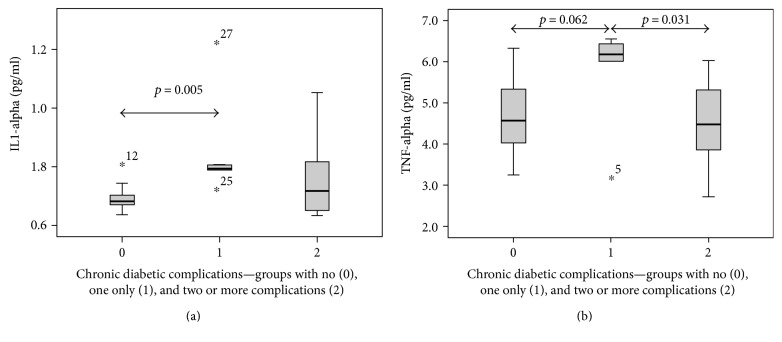
Inflammatory cytokines and chronic diabetic complications. The highest production of IL-1alpha was observed in patients with one chronic diabetic complication which significantly differed from patients without any diabetic complications. TNF-alpha expression levels were similar to those seen for IL-1alpha, but in addition, patients with multiple complications had significantly lower levels of TNF-alpha than those with only one complication. (a) IL-1alpha. (b) TNF-alpha—the highest TNF-alpha production was also observed in patients with one chronic diabetic complication.

Data from our cohort of 28 patients also allowed us to correlate cytokine expression levels with the presence of chronic diabetic complications (i.e., diabetic retinopathy, nephropathy, neuropathy, and diabetic foot). In this context, 11 out of 28 patients (11/28) exhibited none of these complications at the time of sampling (marked as group 0). On the other hand, 6/28 patients suffered from only one complication (group 1) and 11/28 patients with two or more of these chronic diabetic complications comprised the final grouping (group 2).  Figure 2(a) shows the comparison in IL-1alpha production between these three groups, and it was significantly higher in group 1 when compared to that in group 0 (*p* = 0.005). In a similar fashion, the group with only one complication (group 1) exhibited the greatest TNF-alpha production and differed significantly from both the group with multiple complications (group 2, *p* = 0.031) and the group with no complications (group 0, *p* = 0.062) (Figure 2(b)).

**Figure 3:**
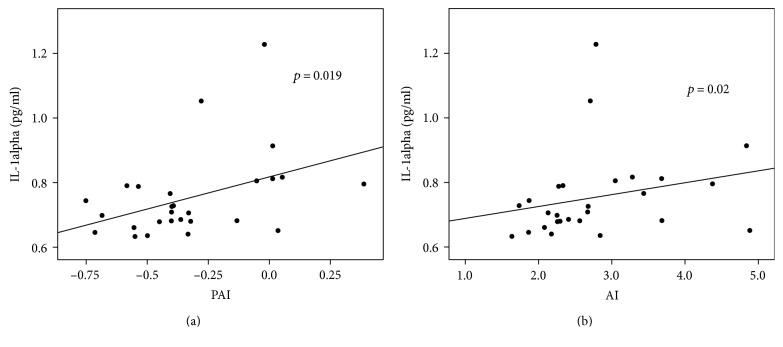
Inflammatory cytokines and calculated lipid indexes. Patients with higher calculated PAI and AI lipid indexes exhibited a significantly higher production of proinflammatory cytokine IL-1alpha (*p* = 0.019 and *R* = 0.449 for PAI and *p* = 0.02 and *R* = 0.446 for AI, resp.). (a) IL-1alpha and PAI (plasma atherogenic index). (b) IL-1alpha and AI (atherogenic index).

Next, the plasma atherogenic index (PAI) which is recommended to be below 0.1 and the atherogenic index (AI), which being below 0.1 indicates the lowest cardiovascular risk, were correlated with IL-1alpha and TNF-alpha cytokine levels. As shown in Figures 3(a) and 3(b), both PAI and AI were significantly positively correlated with IL-1alpha production (*p* = 0.019 for PAI and *p* = 0.02 for AI). There was no correlation with TNF-alpha production observed.

### 3.2. Treg Counts

**Figure 4:**
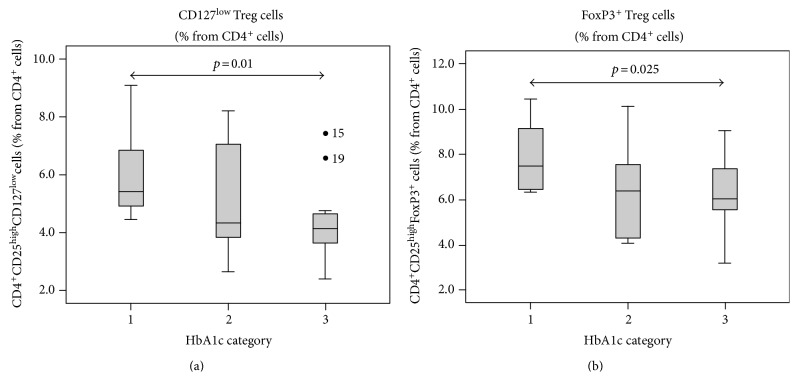
T regulatory cells and HbA1c category. The lowest count of Treg cells was determined in the group of patients with poor diabetes stabilization with the highest HbA1c levels (category 3) and differed significantly from that with the lowest HbA1c, independently whether Treg marker CD127^low^ or FoxP3 was used.

Among many inspected relationships, we found a correlation between Treg counts and diabetes stabilization determined by HbA1c levels, the presence of diabetic neuropathy, lipid spectra, BMI, and IL-6 levels. Notably, in the group with a poor diabetes compensation (group 3, HbA1c > 9% DCCT, see above), we observed significantly lower Treg numbers in comparison to group 1 (perfect or acceptable T1D stabilization). Importantly, this difference was irrespective of whether CD127^low^ (*p* = 0.01, Figure 4(a)) or FoxP3 positivity (*p* = 0.025, Figure 4(b)) was used as the Treg marker.

**Figure 5:**
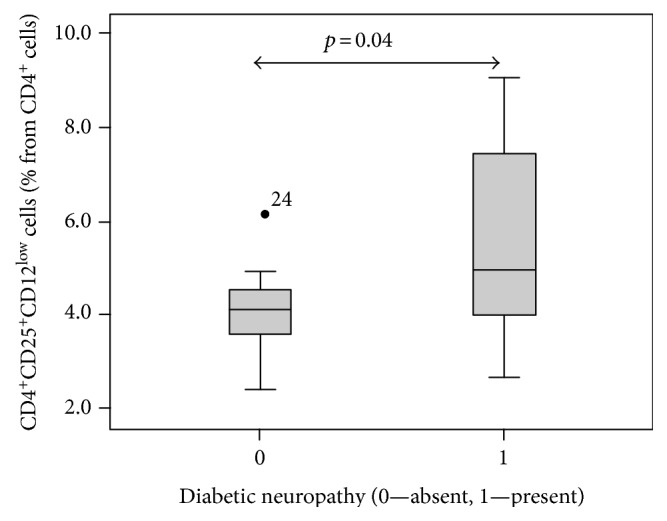
The presence of diabetic neuropathy and CD127^low^ Treg cells. Patients suffering from diabetic neuropathy showed a significantly higher number of CD127^low^ Treg cells than patients with no complications.

In respect to specific diabetic complications, the number of Tregs defined by the CD127 marker (CD4^+^CD25^high^CD127^low^) showed a significant positive correlation with the presence of diabetic neuropathy (*p* = 0.037).  Figure 5 illustrates this finding (the higher CD127^low^ Treg cell numbers in the group with neuropathy, *p* = 0.04). When Tregs were gated as FoxP3 positive cells (CD4^+^CD25^high^FoxP3^+^) or as the combination of CD127^low^ and FoxP3 (CD4^+^CD25^high^CD127^low^FoxP3^+^), positive correlations in regard to the presence of diabetic neuropathy were still apparent but failed to reach significant levels (*p* = 0.369 for CD4^+^CD25^high^FoxP3^+^ and *p* = 0.169 for CD4^+^CD25^high^CD127^low^FoxP3^+^).

**Figure 6:**
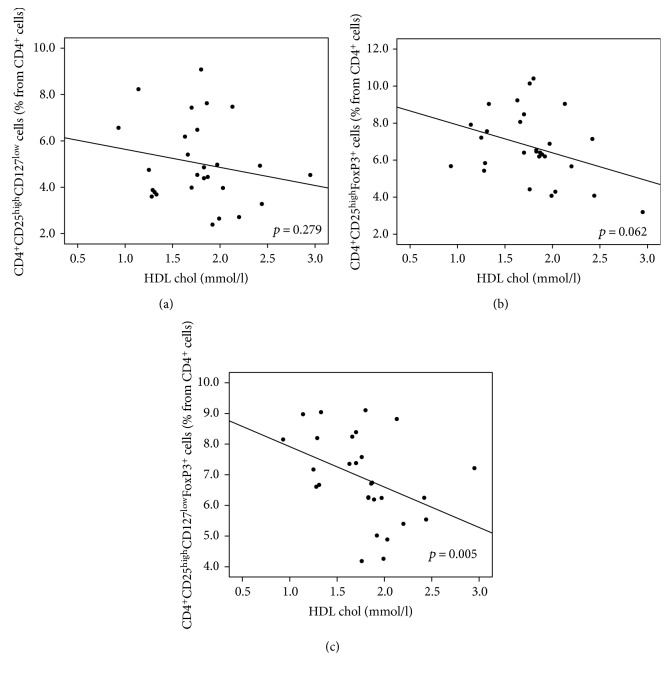
(a–c) The relationship between T regulatory cells and HDL level. Higher level of HDL cholesterol correlated with lower Treg numbers. This correlation was significant in the case of CD127^low^FoxP3^+^ cells (*p* = 0.005, *R* = −0.52). The same trend was observed for both CD127^low^ and FoxP3^+^ Treg cells but failed to reach significant levels (*p* = 0.279 and *R* = −0.216 for CD127^low^ Treg cells and *p* = 0.062 and *R* = −0.357 for FoxP3^+^ Treg cells). (a) CD127^low^ Treg cells (% from CD4^+^cells). (b) FoxP3^+^ Treg cells (% from CD4^+^cells). (c) CD127^low^FoxP3^+^ cells (% from CD4^+^cells).

**Figure 7:**
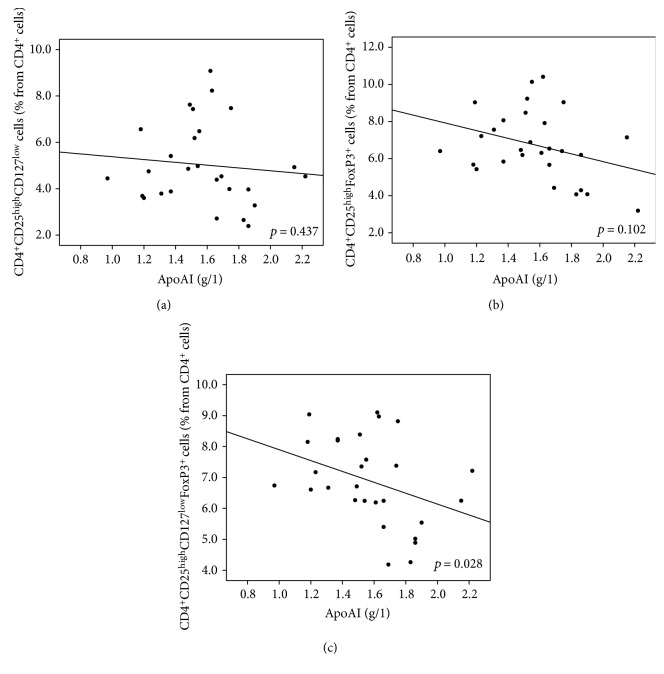
(a–c) The relationship between T regulatory cells and ApoAI. Higher level of ApoAI correlated with lower Treg numbers. This correlation was significant in the case of CD127^low^FoxP3^+^ cells (*p* = 0.028, *R* = −0.416). The same trend was observed for both FoxP3^+^ and CD127^low^ Treg cells but failed to reach significant levels (*p* = 0.102, *R* = −0.315 and *p* = 0.437, *R* = −0.156, resp.). (a) CD127^low^ Treg cells (% from CD4^+^cells). (b) FoxP3^+^ Treg cells (% from CD4^+^cells). (c) CD127^low^FoxP3^+^ Treg cells (% from CD4^+^cells).

**Figure 8:**
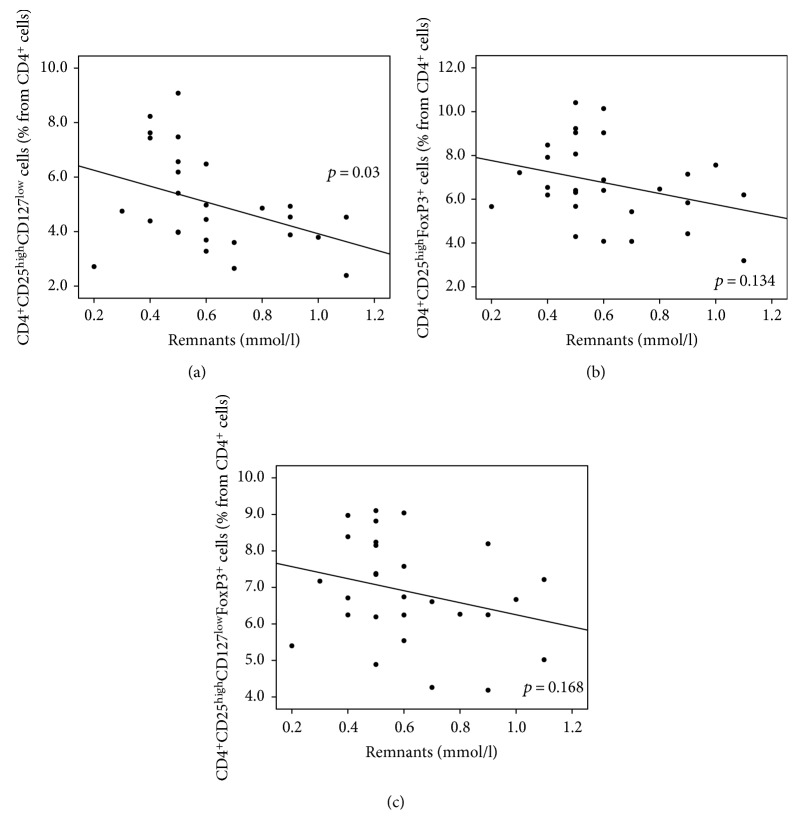
(a–c) The relationship between T regulatory cells and calculated “remnant cholesterol”. Higher calculated remnant cholesterol was correlated to lower Treg numbers. This correlation was significant in the case of CD127^low^ Treg cells (*p* = 0.03, *R* = −0.418). The similar trend was observed for FoxP3^+^ as well as for CD127^low^FoxP3^+^ Treg cells that failed to reach statistical significance (*p* = 0.134, *R* = −0.29 and *p* = 0.168, *R* = −0.268, resp.). (a) CD127^low^ Treg cells (% from CD4^+^cells). (b) FoxP3^+^ Treg cells (% from CD4^+^cells). (c) CD127^low^FoxP3^+^ Treg cells (% from CD4^+^cells).

In contrast to diabetic neuropathy, Treg numbers as defined by CD127 (CD4^+^CD25^high^CD127^low^) and/or FoxP3 markers (CD4^+^CD25^high^FoxP3^+^ or CD4^+^CD25^high^CD127^low^FoxP3^+^) were inversely correlated with HDL cholesterol and ApoAI. These correlations were significant when the combination of both CD127^low^ and FoxP3^+^ markers was used (CD4^+^CD25^high^CD127^low^FoxP3^+^; *p* = 0.005 for HDL and *p* = 0.028 for ApoAI; Figures 6 and 7, resp.). A significant inverse correlation was observed as well as for CD4^+^CD25^high^CD127^low^ Treg cells and the calculated remnant cholesterol (*p* = 0.03). An apparent inverse but nonsignificant relationship for remnant cholesterol and CD4^+^CD25^high^FoxP3^+^ which is CD4^+^CD25^high^CD127^low^FoxP3^+^ Treg cells was present (Figure 8).

**Figure 9:**
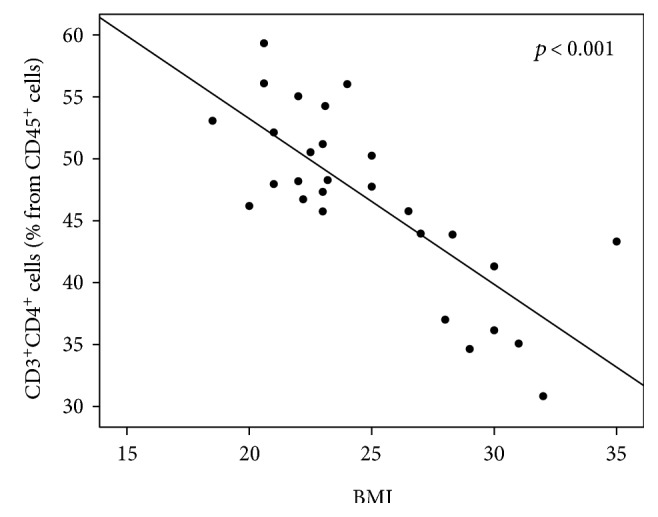
The inverse relationship between CD3^+^CD4^+^ cells (% from CD45^+^cells) and BMI. Patients with higher BMI had significantly lower numbers of CD3^+^CD4^+^ cells (shown as % from CD45^+^ cells). *p* < 0.001, *R* = −0.65.

Interestingly, the total number of CD4^+^ T cells was also inversely correlated with the patient's body mass index (BMI, *p* < 0.001, Figure 9) whereby patients with higher BMI had a lower number of CD3^+^CD4^+^ T cells. In contrast, we found a positive correlation between the number of CD4^+^CD25^high^CD127^low^ Tregs and a patient's BMI, even though this correlation did not reach significance (*p* = 0.087; data not shown).

**Figure 10:**
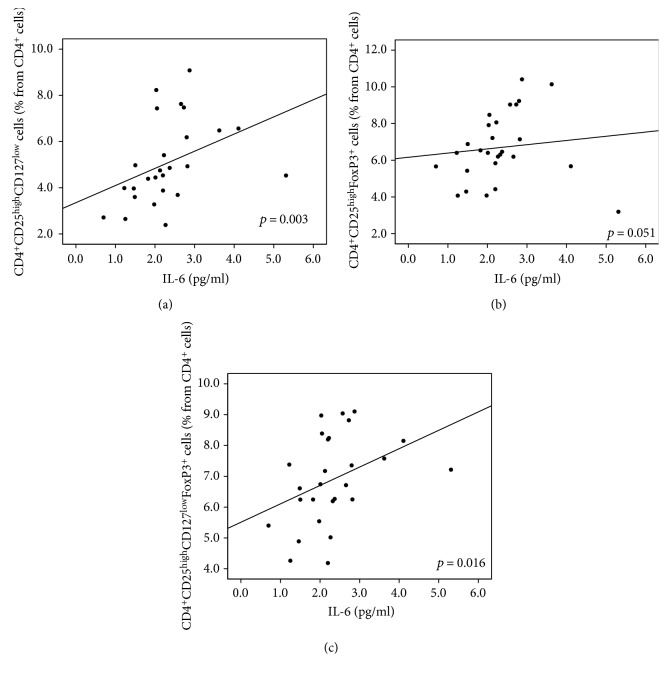
(a–c) T regulatory cells and IL-6. Higher Treg cell count correlated with higher level of proinflammatory cytokine IL-6. This relationship reached significance in the case of CD127^low^ Treg cells (*p* = 0.003, *R* = 0.551) and CD127^low^FoxP3^+^ Treg cells (*p* = 0.016, *R* = 0.458). The same trend with a borderline significance was observed for FoxP3^+^ Treg cells (*p* = 0.051, *R* = 0.379). (a) CD127^low^ Treg cells (% from CD4^+^cells). (b) FoxP3^+^ Treg cells (% from CD4^+^cells). (c) CD127^low^FoxP3^+^ Treg cells (% from CD4^+^cells).

Tregs as defined by the CD127 and/or FoxP3 markers were also positively correlated with IL-6 levels with *p* = 0.003 for CD4^+^CD25^high^CD127^low^, *p* = 0.051 for CD4^+^CD25^high^FoxP3^+^, and *p* = 0.016 for CD4^+^CD25^high^CD127^low^FoxP3^+^ (Figure 10).

### 3.3. Other Metabolic Parameters with Possible Impact to Immune Reactions

#### 3.3.1. Vitamin D

The group with the worst HbA1c levels had significantly lower vitamin D levels (*p* = 0.009 when compared with group 1 and *p* = 0.02 when compared with group 2; Supplementary Figure 2a). Higher glycaemic variability also significantly correlated with lower vitamin D levels (*p* = 0.047, Supplementary Figure 2b). We observed that diabetic patients with LDL cholesterol levels that exceeded the recommended value (LDL cholesterol should be <2.5 mmol/l) had also significantly lower vitamin D levels (*p* = 0.019; Supplementary Figure 3).

**Figure 11:**
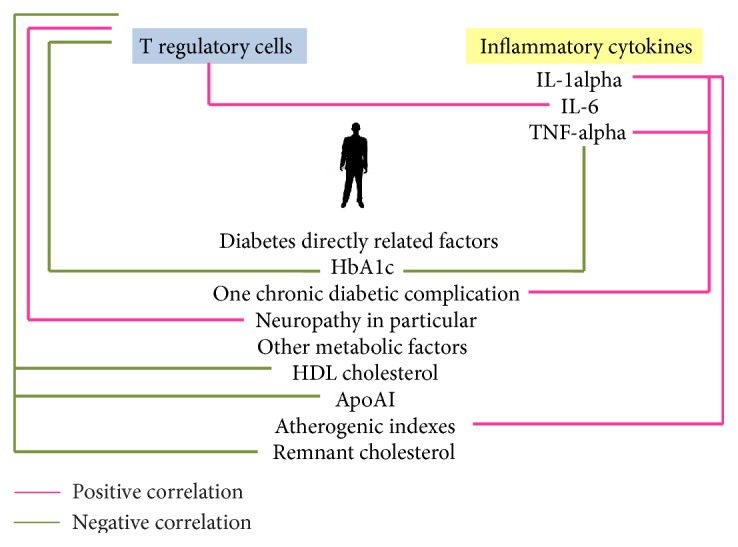
Regulatory T cell count positively correlated with the presence of neuropathy and with IL-6 level. Negative relationship was observed between regulatory T cell count and HbA1c level, HDL, ApoAI, and remnant cholesterol. IL-1alpha level was positively connected with the presence of one chronic diabetic complication (the same stands for TNF-alpha) as well as to calculated atherogenic indexes. We observed a negative relationship between TNF-alpha and HbA1c level.

Figure 11 summarizes the correlations between immune and metabolic parameters revealed in this study.

## 4. Discussion

It is well known that each diabetic patient may eventually become immunologically compromised, particularly if diabetes lasts for a long period of time and is poorly stabilized. In this context, it has been shown that the high frequency of infections among diabetic patients is caused by hyperglycaemia that leads to complex immune dysfunctions, that is, an impairment of neutrophil function, antioxidant system, and humoral immunity [10]. Many diabetes-related comorbidities along with overweight or obesity (if present) may also considerably contribute to immune dysregulation. Currently, there is an emerging consensus among diabetologists on the importance of description and understanding of other aspects of diabetes-associated cellular and genetic changes, such as metabolomics and epigenetics. We are learning how biochemical parameters can imprint our genetic information and eventually understand how these can affect the lives of our newborns via intrauterine transmission. Bidirectional interactions between the immune system and whole-body metabolism is being increasingly recognized, and consequently, a new research field of immunometabolism with a particular focus on immunoregulatory and proinflammatory mechanisms has been recently established [6, 11]. These new trends and our previous research related to this topic inspired us to study the complex interplay between metabolic and immune factors of T1D patients in general and, in this context, the effect of T1D mothers on their offspring, in particular.

The parameter that is widely used to monitor lasting hyperglycaemia is the level of HbA1c. It reflects the glycaemic status of an individual for approximately the last 3 months [12], and not surprisingly, this factor exerts important correlations with several immune and metabolic parameters. For example, it has been reported that hyperglycaemia itself has immunosuppressive and proinflammatory effects [10]. Consistent with a recent report [13], we also observed a lower number of Treg cells in patients with higher HbA1c levels. However, while the HbA1c level is a very useful parameter for evaluating diabetes stabilization, it does not reflect the glycaemic variability which is increasingly used as an important marker in the development of chronic diabetic complications that determines a patient's long-term prognosis [1]. We failed to correlate glycaemia standard deviation (SD), considered to be a basic parameter that represents glycaemia variability [14], with immunoregulatory functions and/or proinflammatory cytokine production in our study. On the other hand, glycaemia SD correlated with lipid spectra parameters, insulin dose (data not shown), and vitamin D which may indicate the rather indirect effect of glycaemic variability to immune parameters. Thus, our data support the view that changes in immune parameters and occurrence of chronic diabetes complications reflect the total time spent in hyperglycaemic state.

One interesting finding is that both poor HbA1c values and higher glycaemic variabilities were associated with lower vitamin D levels. In this sense, a majority of samples used in our study were collected in the spring/summer season (April–September) when vitamin D levels are not dramatically affected by the low sun exposure of our patients. Vitamin D is important for proper immune function, and there are reports that low vitamin D levels are associated with the development of autoimmune conditions including diabetes [15]. Vitamin D probably affects negative selection in the thymus, effector Th1 and Th17 pathogenesis, the responsiveness to extrinsic cell death signals, FoxP3^+^CD4^+^ Tregs and CD4^+^ T-regulatory cell type 1 (Tr1) functions, and the Th1-Tr1 switch [15]. However, our results suggest that poorer diabetes stabilization may at least partially contribute to reduced vitamin D levels that have frequently been reported in diabetic individuals [16]. Moreover, there are reports that low vitamin D levels (which are highly prevalent worldwide) are associated with metabolic syndrome. Reciprocally, it is well known that vitamin D, via various mechanisms, can affect glycaemic control [17, 18]; therefore, the functional link between diabetes and vitamin D levels may truly be bidirectional.

Surprisingly, a poorer glycaemic control was associated with lower TNF-alpha levels, which was quite opposite to the situation that we expected. The greatest TNF-alpha production as well as IL-1alpha production was observed in patients who exhibited one chronic diabetic complication. These cytokines are mainly produced by activated macrophages, but neutrophils, epithelial cells, and endothelial cells can also contribute. IL-1alpha possesses metabolic, physiological, and haematopoietic activities and plays a central role in the regulation of immune responses. For example, it affects T-helper cells causing the induction of IL-2 secretion and expression of IL-2 receptors [19]. However, there is data which suggests that IL-1alpha could be a cause of the development of chronic diabetic complications because of certain genetic variants that were shown to be positively associated with diabetic nephropathy [20]. This observation may reflect the fact that the processes underpinning the development of multiple chronic diabetic complications were still active in these participants. In contrast, in patients with multiple complications, we observed an overall exhaustion and improper activation of the innate immune mechanisms which were reflected by lower IL-1alpha levels.

We also observed a positive correlation between chronic diabetic neuropathy and Treg counts which is more difficult to explain. Such an association is likely to be indirect as defective immunoregulation by Tregs, compensated by their higher numbers, would compromise nerve protection, but we lack data to support this view [21].

Interestingly, higher IL-6 levels were associated with higher Treg numbers as defined by either of the two employed Treg markers (CD127^low^ and/or FoxP3) despite the fact that the presence of IL-6 inhibits FoxP3 function by diverting Treg differentiation in favour of the Th17 phenotype [22]. These findings may indicate that imbalances between FoxP3 and retinoic acid-related orphan receptor gamma t (ROR*γ*t), the key transcription factor in Th17 cells, could be present in T1D. Such imbalances could be very important because the equilibrium between these two molecules determines CD4 T cell fate and the type of subsequently generated immune responses. The suspicion that the Treg/Th17 balance is very important for the development of T1D is also supported by our previously published study [23]. Moreover, it has been previously proposed that the development of Th1/Th17 plasticity may serve as a biomarker of disease progression from beta cell autoantibody positivity to clinically apparent type 1 diabetes [24].

As far as the impact of other nonimmune factors is concerned, it is known that T1D may acquire certain “T2D” (type 2 diabetes) or metabolic syndrome “features” as a patient ages, primarily when a patient's body weight increases; hence, these features may interfere with the patient's immunoreactivity. This assumption led us to concentrate on other factors related to metabolic syndrome in addition to insulin resistance. Our results also suggest that neither the presence of hypertension nor insulin resistance are linked to studied immune and metabolic parameters; one exception is being the correlation between hypertension and calculated remnant cholesterol (data not shown). In contrast, body weight was negatively correlated with the total T-helper cell count, which is consistent with recent reports which showed reductions in specific circulating lymphocyte populations in patients who are at risk of developing T2D [25].

An emerging relationship between the Treg count on one hand and HDL and ApoAI levels (ApoAI is the main protein component of HDL) on the other hand could be of clinical importance. There is an evidence of a skewed balance between anti- and proinflammatory T cell subsets in T2D patients, and it has been reported that HDL strongly affects T cell polarization [26]. Moreover, ApoAI has been found to modulate regulatory T cells in autoimmune LDLr^−/−^ and ApoAI^−/−^ mice [27]. Thus, our data provides additional evidence supporting direct links between these lipid factors and the number, differentiation, and function of Tregs in humans.

It is difficult to explain the level of inconsistency of our results when different Treg markers are used, that is, CD127^low^ versus FoxP3^+^. A group of Japanese researchers noted that CD4^+^CD25^+^CD127^low^ T cells isolated from IPEX (Immune dysregulation, Polyendocrinopathy, Enteropathy, X-linked) patients also exhibited appreciable suppressive activity, although less than that exhibited by Treg cells from healthy controls. Their results suggest that genetically altered FoxP3 can drive the generation of functionally immature Treg cells, but intact FoxP3 is necessary for the generation of fully functional Tregs. However, T1D patients should express fully functional FoxP3. On the other hand, FoxP3 was identified as an important epigenetic modifier, and accumulating evidence suggests an intriguing functional convergence between FoxP3 and inhibitors of histone deacetylases [28, 29]. In addition, epigenetic changes may affect FoxP3 gene expression itself. Thus, due to subtle changes related to the expression of FoxP3 and CD127 in Tregs from T1D patients, discrepancies in defining Tregs using either of these markers could reveal differences. Finally, a third possibility which may explain these inconsistent results could be the fact that FACS-mediated FoxP3 analysis requires cell permeabilization, while CD127 does not. Therefore, at this point, we can only speculate if the observed discrepancy is due to the alteration of a specific Treg subset or if it is the result of Treg manipulation and staining [28, 29].

## 5. Conclusion

Our results show an intricate relationship between glycaemic, lipid, and immune parameters in T1D female patients. We conclude that one important factor that negatively affects immune parameters such as Treg counts, TNF-alpha production, and vitamin D levels is the total time spent in the hyperglycaemic state reflected in HbA1c values. On the other hand, glycaemia variability even though it seems not to have direct impact to studied immune parameters highly correlated with other metabolic factors including vitamin D and thus can be of potential clinical and prognostic value. Moreover at this junction, it is difficult to determine the cause and effect relationships between multiple nonimmune factors. Their associations suggest that preventing T1D patients from increasing their body weight and from developing dyslipidemia could be beneficial for a more favourable long-term prognosis from many different aspects. Our data also suggests that complementing glycaemic data with lipid clinical parameters can provide clinicians with a more comprehensive view on the immunometabolism of T1D patients and their long-term prognosis. It would be important now to extend these findings to understand if and how these complex metabolic features of T1D female patients affect the immunity and fate of their children.

## Supplementary Material

Supplemental Figure 1. displays the gating strategy. The gate number 1 represents CD45^+^ lymphocytes. From this gate, Th lymphocytes were gated as CD3^+^CD4^+^ cells (Gate #2). Gates #3, #4 and #5 represent cells carrying following marker combinations: CD4^+^CD25^high^ (Gate #3), CD4^+^CD25^high^ CD127^low^ (Gate #4), CD4^+^CD25^high^ FoxP3^+^ (Gate #5), respectively. Finally Gate #6 represents CD4^+^CD25^high^CD127^low^FoxP3^+^ cells. The gated cell population is indicated by demarcation line or circle (for example Th lymphocytes in Gate #2 are marked by a circle). It is of note that FoxP3 positivity among CD25^+^ cells was always determined by a costaining analysis with anti-FoxP3 versus anti-CD25 (see the dot plot with the Gate #5). It shows the proportion and intensity of CD25^+^ cells that are also FoxP3 positive. As clearly demonstrated in Gate 5, many CD25^low/intermediate^ cells are also FoxP3^+^. Rather than using an arbitrary gating for CD25^high^ cells which would likely ignore and/or eliminate a tangible population of FoxP3^+^ cells from our analysis, we used this staining as a guiding principle to define CD25^+^ FoxP3^+^ cells. Using this gating strategy, >85% of CD25^+^ cells were also Foxp3^+^. Our data presented in the Suppl. Fig 1., also show that using the same and unbiased gating for CD25^+^ CD127^−/low^ cells (Gate #4) demarcates a subpopulation from which >85% of cells are FoxP3^+^ (Gate #6). However, as many CD25^+^cells are also FoxP3^−^, they likely accounts for discrepancies when CD127 versus Foxp3 (or their combination) are used to gate on Tregs (see the result section). Supplemental Figure 2. The worst diabetes stabilization with the worst HbA1c (cathegory 3) was linked to lower vitamin D levels. The difference was significant when these patients were compared to well stabilized patients (cathegory 1) as well as to patients with intermediate HbA1c levels (cathegory 2). Moreover, higher glycaemia variability was connected to lower vitamin D levels (p=0.047, R=-0.418). Supplemental Figure 3. Patients with LDL cholesterol exceeding the recommended levels displayed significantly lower vitamin D levels compared to patients with normal LDL cholesterol. Supplemental Table 1. Laboratory values: patient metabolic characterisations.

## Figures and Tables

**Table 1:** Clinical characteristics of the study population.

	Median	Range
Age (years)	35	22–42
Diabetes duration (years)	12	6–27
BMI	23.1	18.5–35
Insulin units per kg	0.7	0.4–1.4

	Present	

Autoimmune thyroiditis	21/28	
Other autoimmune disease	2/28	
Hypertension	8/28	
Dyslipidaemia	9/28	
	But only 1 treated	
Diabetic retinopathy	12/28	
Diabetic neuropathy	11/28	
Diabetic nephropathy	8/28	
Diabetic foot syndrome	1/28	
Multiple chronic complications (2 and more)	11/28	

**Table 2:** Definitions of the grouping variables.

Grouping variable	Groups	Group definition
HbA1c	1 (perfect or acceptable T1D stabilization)	HbA1c < 7.5% DCCT
	2 (not very well stabilized)	HbA1c according to DCCT in the range of 7.5 to 9.0%
	3 (badly compensated)	HbA1c > 9.0% DCCT

Glycaemic variability	1 (acceptable glycaemia variability)	Glycaemia SD ≤ 1/3 of mean glycaemia
	2 (high glycaemia variability)	Glycaemia SD > 1/3 of mean glycaemia

eGDR	RG	eGDR < 7.5
	NRG	eGDR ≥ 7.5

The presence of chronic diabetic complications	0 (no complication)	
	1 (one chronic diabetic complication present)	
	2 (two or more chronic diabetic complications present)	

LDL cholesterol	0 (low and normal values)	LDL < 2.5 mmol/l
	1 (increased levels)	LDL ≥ 2.5 mmol/l

Hypertension	0 (low and normal values)	Blood pressure < 130/80
	1 (increased blood pressure values or treatment with antihypertensive drugs)	

## Abbreviations

Apo: Apolipoprotein
BMI: Body mass index
Chronic diabetic complication category 0: No complication present
Chronic diabetic complication category 1: One chronic diabetic complication present
Chronic diabetic complication category 2: Two or more chronic diabetic complications present
DCCT: Diabetes Control and Complication Trial
eGDR: Whole-body-estimated glucose disposal rate
GV: Glucose variability
HbA1c group 1: Perfect or acceptable T1D stabilization
HbA1c group 2: Not well stabilized
HbA1c group 3: Badly compensated patients
HbA1c: Glycosylated haemoglobin
HDL: High-density lipoproteins
IU: Insulin units
LDL category 0: Low and normal values
LDL category 1: Increased levels
LDL: Low-density lipoproteins
n.s.: Nonsignificant
NRG: Nonresistance group
RG: Resistance group
SD: Standard deviation
T1D: Type 1 diabetes.
