# Supplementary material for: Not Only Glycaemic But Also Other Metabolic Factors Affect T Regulatory Cell Counts and Proinflammatory Cytokine Levels in Women with Type 1 Diabetes
Source: J Diabetes Res. 2017 May 3;2017:5463273. doi: 10.1155/2017/5463273 (PMC5434466; doi:10.1155/2017/5463273)
Supplement: Supplementary file 1 — Supplemental Figure 1. displays the gating strategy. The gate number 1 represents CD45+ lymphocytes. From this gate, Th lymphocytes were gated as CD3+CD4+ cells (Gate #2). Gates #3, #4 and #5 represent cells carrying following marker combinations: CD4+CD25high (Gate #3), CD4+CD25high CD127low (Gate #4), CD4+CD25high FoxP3+ (Gate #5), respectively. Finally Gate #6 represents CD4+CD25highCD127lowFoxP3+ cells. The gated cell population is indicated by demarcation line or circle (for example Th lymphocytes in Gate #2 are marked by a circle). It is of note that FoxP3 positivity among CD25+ cells was always determined by a costaining analysis with anti-FoxP3 versus anti-CD25 (see the dot plot with the Gate #5). It shows the proportion and intensity of CD25+ cells that are also FoxP3 positive. As clearly demonstrated in Gate 5, many CD25low/intermediate cells are also FoxP3+. Rather than using an arbitrary gating for CD25high cells which would likely ignore and/or eliminate a tangible population of FoxP3+ cells from our analysis, we used this staining as a guiding principle to define CD25+ FoxP3+ cells. Using this gating strategy, >85% of CD25+ cells were also Foxp3+. Our data presented in the Suppl. Fig 1., also show that using the same and unbiased gating for CD25+ CD127−/low cells (Gate #4) demarcates a subpopulation from which >85% of cells are FoxP3+ (Gate #6). However, as many CD25+cells are also FoxP3−, they likely accounts for discrepancies when CD127 versus Foxp3 (or their combination) are used to gate on Tregs (see the result section). Supplemental Figure 2. The worst diabetes stabilization with the worst HbA1c (cathegory 3) was linked to lower vitamin D levels. The difference was significant when these patients were compared to well stabilized patients (cathegory 1) as well as to patients with intermediate HbA1c levels (cathegory 2). Moreover, higher glycaemia variability was connected to lower vitamin D levels (p=0.047, R=-0.418). Supplemental Figure 3. Patient [file 5463273.f1.docx]

**SUPPL. TABLE 1: Laboratory values: patient metabolic characterisations**

|  |  | **eGDR**  **mg.kg^-1^.min^-1^** | **Cholesterol (total)**  **mmol/l** | **LDL choleste-rol**  **mmol/l** | **HDL cholesterol**  **mmol/l** | **Triglycerides**  **mmol/l** | **ApoAI**  **g/l** | **ApoB**  **g/l** | **Uric acid**  **µmol/l** | **C peptide**  **pmol/l** |
| --- | --- | --- | --- | --- | --- | --- | --- | --- | --- | --- |
| **Normal laboratory**  **values** | | ≥ 7.5 | 3.4-5 | 1.5-3 | 0.72-2.69 | 0.4-1.98 | 1.39-1.8 | 0.65-1.05 | 208-434 | 370-1470 |
| **Median** | | 10.5 | 4.6 | 2.1 | 1.8 | 0.76 | 1.58 | 0.7 | 197 | 12 |
| **Minimum** | | 3.5 | 3.6 | 0.9 | 0.9 | 0.4 | 1.0 | 0.4 | 125 | 0.0 |
| **Maximum** | | 12.,6 | 6.3 | 4.1 | 3.0 | 3.1 | 2.2 | 1.6 | 423 | 429 |
