# Supplementary figures and images for: Not Only Glycaemic But Also Other Metabolic Factors Affect T Regulatory Cell Counts and Proinflammatory Cytokine Levels in Women with Type 1 Diabetes
Source: J Diabetes Res. 2017 May 3;2017:5463273. doi: 10.1155/2017/5463273 (PMC5434466; doi:10.1155/2017/5463273)

## Slide 1
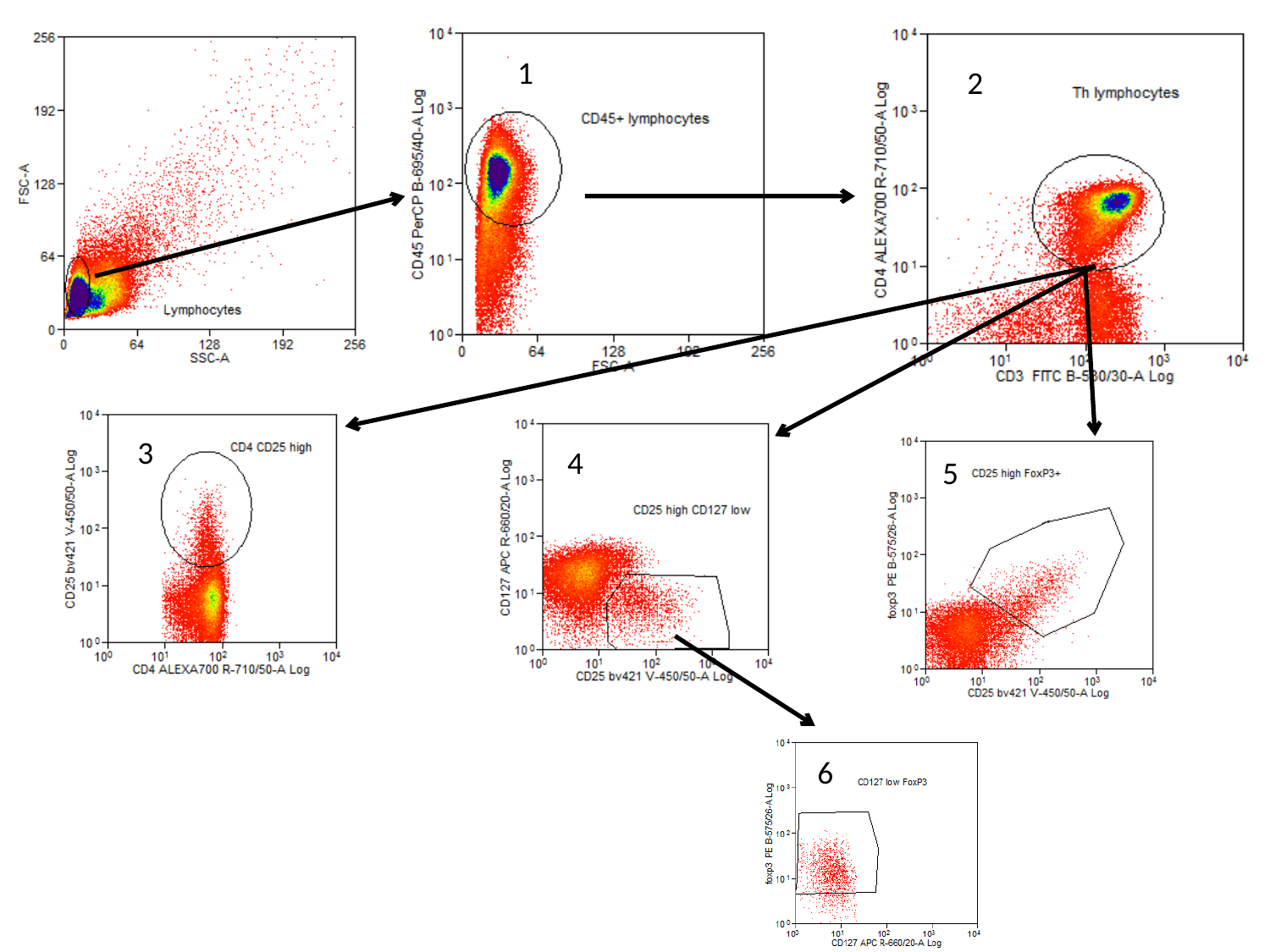

1
2
3
4
5
6

Supplement: Supplementary file 2 [file 5463273.f2.ppt]

## Slide 1
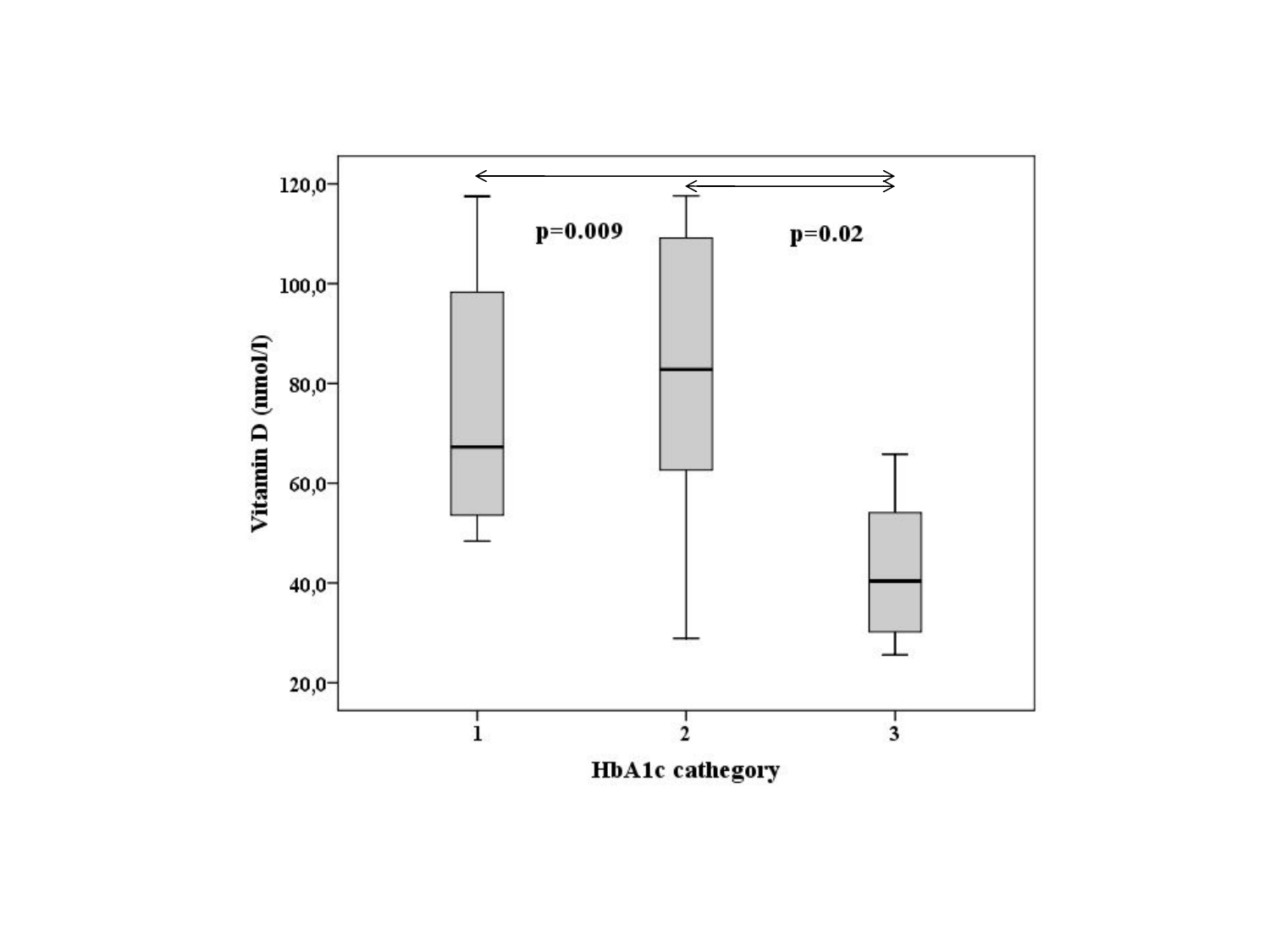

## Slide 2
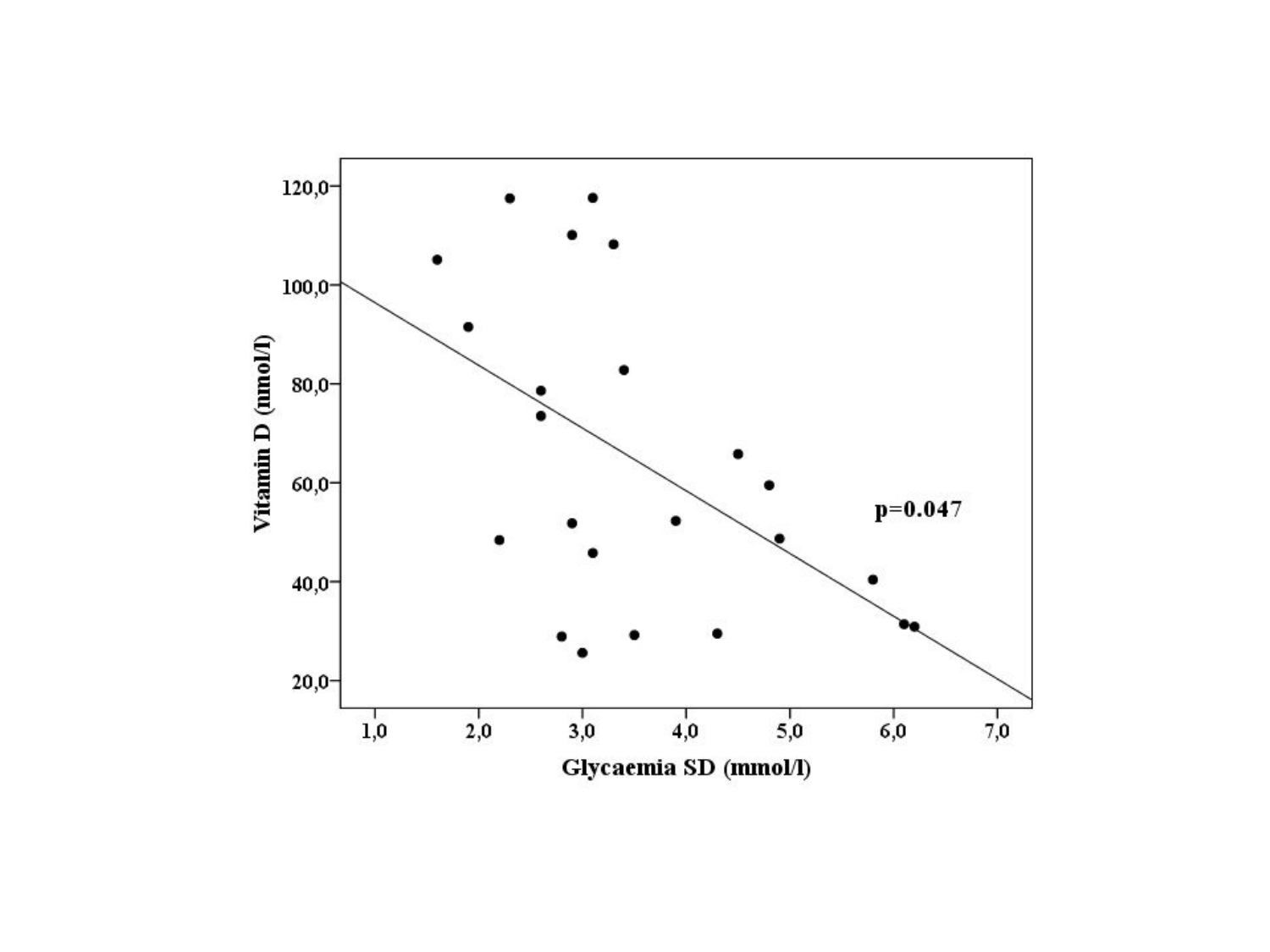

Supplement: Supplementary file 3 [file 5463273.f3.ppt]

## Slide 1
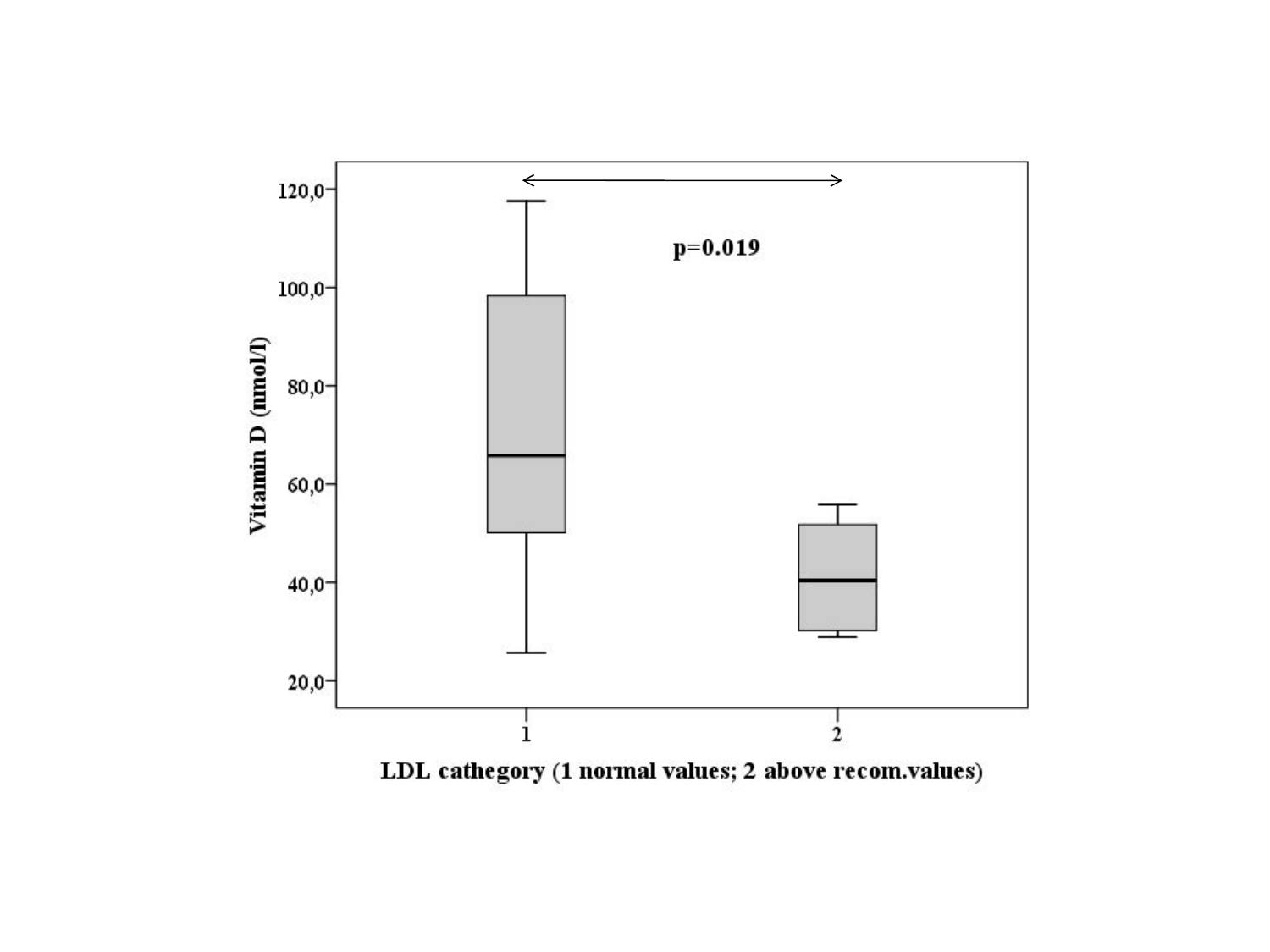

Supplement: Supplementary file 4 [file 5463273.f4.ppt]
